# COVID-19 Experiences and Health-Related Implications: Results From a Mixed-Method Longitudinal Study of Urban Poor Adolescents in Shanghai

**DOI:** 10.1016/j.jadohealth.2022.03.016

**Published:** 2022-07

**Authors:** Mengmeng Li, Chunyan Yu, Xiayun Zuo, Celia Karp, Astha Ramaiya, Robert Blum, Caroline Moreau

**Affiliations:** a Department of Population, Family and Reproductive Health, Johns Hopkins Bloomberg School of Public Health, Baltimore, Maryland; b NHC Key Laboratory of Reproduction Regulation (Shanghai Institute of Planned Parenthood Research), Fudan University, Shanghai, China; c Soins Primaires et Prévention. Inserm U1018, Center for Research in Epidemiology and Population Health (CESP), Villejuif, France

**Keywords:** COVID-19 pandemic, SARS-CoV-2, COVID-19 impacts, Adolescent health, Mental health, Gender inequality

## Abstract

**Purpose:**

This analysis aimed to investigate gender differences in adolescents’ concerns and the health implications of COVID-19.

**Methods:**

We used two rounds of the Global Early Adolescent Study (GEAS) collected in Shanghai in 2018 and 2020. We analyzed data from 621 adolescents, comparing boys’ and girls’ concerns about COVID-19 and examining trends in general health and mental health by sex between the pre-COVID-19 and COVID-19 periods. Changes in health indicators over time were assessed using generalized estimating equation (GEE) models.

**Results:**

Adolescent girls reported more health concerns (52.0% vs. 42.7%) and educational concerns (61.0% vs. 46.3%) than boys, whereas boys expressed more worries about the economic consequences of COVID-19 (32.9% vs. 25.4%). Changes in health-related outcomes during the pandemic compared to the prepandemic era differed by sex and varied by COVID-related experiences. Boys reported improved overall health (OR: 1.54, 95% CI: 1.00, 2.35) in the COVID-19 period relative to the pre-COVID-19 period. Such improvements were only observed among boys who reported no family economic hardships (OR: 2.10, 95% CI: 1.24, 3.58). We found no significant change for girls (OR: 1.14, 95% CI: 0.83, 1.55), regardless of COVID-19 economic impacts. In contrast, girls reported increased anxiety (OR: 1.63, 95% CI: 1.09, 2.45), especially among those who were concerned about their academic performance (OR: 1.85, 95% CI: 1.16, 2.97). Boys experienced no such increase (OR: 0.92, 95% CI: 0.55, 1.54), regardless of their education concerns.

**Discussion:**

Adolescents’ COVID-19 experiences are highly gendered and result in increased health inequalities, with greater mental health implications for girls.

**Implications and Contribution**

COVID-19 impacts on health outcomes vary according to adolescents’ concerns about or hardships imposed by the pandemic. Thus, these findings emphasize the need for social support tailored to meet the distinct COVID-19 experiences of adolescents from low-resource communities in Shanghai, China, to buffer the negative gendered consequences of the pandemic.

COVID-19 was first reported in Wuhan, Hubei province, in December 2019, spreading to all major cities in China, including Shanghai, by January 2020 [1]. Stringent public health restrictions were implemented nationwide in early February, leading to rapid and effective containment of the first wave of the epidemic. Restrictions remained in effect for several months, with schools moving to online teaching through late April 2020.

As with past epidemics, COVID-19 amplified existing ethnic and social inequalities globally, disproportionately affecting socially disadvantaged populations, who face a greater risk of morbidity and mortality [[2], [3], [4]]. The pandemic’s effects have been devastating among poor urban communities throughout the world, which grapple with limited access to resources and disposable income to sustain COVID-19-related lockdowns [[5], [6], [7]]. In addition, the closure of schools may have increased educational disparities due to lack of internet access while also limiting social interactions among adolescents [8,9]. Such impacts were seen in sub-Saharan Africa during the Ebola epidemic, and school closure increased school dropout, child labor, violence against children, teenage pregnancies, and socioeconomic disparities [10].

There are also reports from around the world of growing social disparities related to COVID-19 intersecting with rising COVID-related gender inequalities [[10], [11], [12]]. Evidence from past public health emergencies indicates that women and girls face increased gender-based violence and loss of educational and economic opportunities [13] while also assuming more caretaking and household duties than in prepandemic eras. These gender patterns have been reported in Europe and the United States during COVID-19 lockdowns [[14], [15], [16]] and may have contributed to poor mental health among women and girls [15,17,18] at a time when psychosocial supports were limited. The rising division of gender roles in the context of limited resources is likely to have longer-term implications for health disparities [11].

While a number of experts have suggested that COVID-19 may exacerbate gender inequalities [9,19], to date, there has been little attention paid to the implications for adolescents. For bridging this gap, our study compares young boys’ and girls’ early concerns related to the pandemic in urban poor communities in Shanghai and how these concerns inform their health and wellbeing.

## Methods

This mixed-method study draws on an existing cohort of young adolescents from the Global Early Adolescent Study (GEAS) in Shanghai. The GEAS is a multisite study exploring gender socialization and its implications for adolescent health in 11 low-income urban communities across five continents (https://www.geastudy.org) [20]. In Shanghai, GEAS is conducted in the northern part of the Jing'an District, home to the largest proportion of urban poor and internal migrants. In 2018, 1,760 adolescents 10–14 years were recruited from three large public secondary schools. All eligible students in grades 6–8 were invited to participate after providing their parents’ informed consent and separately providing their own assent to participate. Adolescents were invited to self-complete an annual survey on tablets in school, soliciting information about their social context (e.g., family, peers, school, and neighborhood), their agency and perceptions of gender norms, as well as their health-related behaviors and outcomes (pubertal development, body comfort, mental health, sexual health, interpersonal violence). The GEAS survey was developed following formative mixed-pre-COVID method research in 15 sites across five continents, and the validated instruments are available for public use (https://www.geastudy.org).

This mixed-method study builds on the GEAS platform in Shanghai and employs a concurrent triangulation design to investigate trends in adolescent health-related to COVID-19 experiences, with an emphasis on quantitative results supported by qualitative findings. The qualitative and quantitative COVID-19 study was approved by the institutional review board at the Shanghai Institute of Planned Parenthood Research in Shanghai (No. PJ2020-26).

### Quantitative

#### Data and analytic sample

The quantitative study included a cohort of 1,760 adolescents ages 10–14 years, recruited from three public schools located in Jing'an's two less developed subdistricts in 2017. Adolescent assent and parental consent were obtained in each annual round of data collection. For this study, we used two rounds of GEAS data completed in late 2018 (pre-COVID-19) and 2020 (during the pandemic). Both rounds were self-administered in school using computer-assisted self-interview and took on average 40 minutes to complete. Both rounds collected similar information, including respondents’ perceived overall health and mental health status. The 2020 survey took place in January and June, as it was interrupted by the COVID-19 lockdown: 167 respondents completed the survey in January, while 1,121 completed the survey in June. A COVID-19 module, which took an additional 15 minutes, was added to the June survey to explore adolescents’ knowledge, attitudes, and practices related to COVID-19 and the economic, educational, and health implications of the outbreak. Altogether, 715 adolescents completed the COVID-19 module; however, 680 respondents were successfully linked to previous GEAS data. We excluded 59 respondents who were missing more than 15% of responses, resulting in 621 adolescents in our postlockdown sample. We refer to the 2018 survey as “pre-COVID” and the 2020 postlockdown survey as the “COVID-19” (n = 621) samples. Outcome measures did not differ between adolescents included and excluded in the sample, except for generalized anxiety disorder (GAD). In addition, adolescents included in the analytic sample were more likely to live with both parents at the time of COVID-19 interview (85.9% vs. 75.6%, *p* = .04), were less likely to have siblings (76.7% vs. 81.2%, *p* = .01), and more likely to be from the poorest families (lowest wealth tertile, 28.0% vs. 15.6%, *p* = .03).

#### Measures

We considered several measures to explore adolescents’ perceptions of COVID-19 threats and their concerns about the social and educational implications of the pandemic. Perceptions of COVID-19 threats were derived from four questions focused on worries, stressors, and avoidance behaviors related to the virus [21]. Exploratory factor analysis indicated the four items loaded on a single factor, labeled as COVID-19 concerns (polychoric ordinal alpha = 0.81). We created a mean score of adolescents’ COVID-19 concerns by averaging responses to the four items and created a dichotomized variable at the mean cutoff. The impact of COVID-19 on household economic security and education included measures of household employment loss, food shortages, difficulty affording necessities, and concerns about school grades and progression. Adolescents’ health behaviors and outcomes during COVID-19 included several dimensions: sleeping patterns during the lockdown, average hours of daily physical activity in the last week, perceived overall health (excellent, good, fair, or poor), depressive symptoms, and generalized anxiety disorder (GAD). We removed 45 outliers of hours of sleep (≤2 or ≥16 hours) from the analysis. Depressive symptoms were assessed via three items used in previous rounds of GEAS (polychoric ordinal alpha = 0.84) and the GAD-7 instrument (polychoric ordinal alpha = 0.97) [18]. We dichotomized the anxiety measure by combining moderate and severe anxiety into a single category. Perceived health, depressive symptoms, and anxiety measures were also collected in the pre-COVID-19 survey, allowing a pre-COVID-19 to a COVID-19 trends analysis.

#### Analysis

We first conducted a descriptive analysis of the COVID-19 sample, examining adolescents’ concerns about the pandemic and distributions of economic, social, and educational implications of COVID-19. We also examined key health indicators (i.e., sleep patterns, physical activity, perceived health, depressive symptoms, anxiety) and tested for differences between boys and girls using the Student t-test and Chi-square tests, as appropriate. Next, we examined changes in health indicators between pre-COVID-19 and COVID-19 surveys using generalized estimating equation (GEE) models. We tested for interactions to evaluate if health changes during COVID-19 differed by COVID-19 experiences, including pandemic concerns, school concerns, and economic hardship. We included school fixed effects to account for differential trends by the school environment. Missing values on COVID-19-related variables were marginal (<2.5%), so we applied simple imputation using the most frequent category of each variable to replace missing values. All analyses were stratified by sex and conducted using Stata/SE 15.1 (StataCorp LLC, College Station, TX) and R Version 4.0.5.

### Qualitative

The qualitative data were based on four sex-specific focus group discussions (FGDs) conducted in June 2020 among adolescent boys and girls aged 14–18 years residing in the same subdistricts as the GEAS cohort. Adolescents aged 14–15 years were recruited from the GEAS cohort, while adolescents aged 16–18 were recruited from a high school in the same subdistrict. After providing assent and parental consent (for minors), adolescents were invited to participate in school-based FGDs in June 2020, with appropriate social distancing and protective measures. FGDs, which took on average 90 minutes, were facilitated by young, trained researchers who followed an interviewer guide exploring adolescents’ understanding of and experiences related to COVID-19 prevention, trusted information sources, the impact of COVID-19 lockdowns on relationships, challenges, and opportunities related to remote learning, coping strategies, and support needed during the lockdown.

The FGDs were recorded, transcribed verbatim, and translated with quality checks of all FGD translations. We conducted a thematic analysis based on deductive and inductive coding. All qualitative codes were organized into matrices by sex and age to explore gender and age patterns in young people’s experiences and coping strategies during the lockdown. Deductive codes included the overarching themes outlined above. Thereafter, inductive codes were developed based on adolescents’ responses provided. Qualitative analyses were conducted using Atlas.ti 9.0.

## Results

Table 1 presents the characteristics of the COVID-19 sample (n = 621). On average, participants were 14.4 years old. More than 80% lived with both parents, and nearly all adolescents (96.0%) were at their age-appropriate grade level or above.

**Table 1:** Study population demographic characteristics of COVID survey participants from the GEAS study in Shanghai, China

	Pre-COVID	COVID		
	Overall (N = 621) % (95% CI)	Overall (N = 621) % (95% CI)	Boys (N = 308) % (95% CI)	Girls (N = 313) % (95% CI)
Age (mean (95% CI))	12.91 (12.85–12.97)	14.38 (14.33–14.44)	14.39 (14.31–14.47)	14.37 (14.30–14.45)
11–14	99.52 (98.59–99.90)	54.43 (50.42–58.40)	54.22 (48.48–59.88)	54.63 (48.94–60.24)
15–17	0.48 (0.10, 1.41)	45.57 (41.60–49.58)	45.78 (40.12–51.52)	45.37 (39.76–51.06)
Education attainment				
Below grade level	1.29 (0.58–2.52)	4.03 (2.62–5.89)	5.19 (3.00–8.30)	2.88 (1.32–5.39)
At or above grade level	98.55 (97.27–99.34)	95.97 (94.11–97.38)	94.81 (91.70–97.00)	97.12 (94.61–98.68)
N/A	0.16 (0.00–0.89)	-	-	-
Household				
Both parents	85.19 (82.14–87.89)	80.84 (77.52–83.86)	81.82 (77.05–85.96)	79.87 (75.00–84.17)
One parent only	10.95 (8.60–13.67)	7.73 (5.75–10.12)	6.49 (4.01–9.85)	8.95 (6.03–12.67)
Grandparents (no parents)	3.54 (2.23–5.31)	3.86 (2.49–5.70)	3.57 (1.80–6.30)	4.15 (2.23–7.00)
Other (no parents and grandparents)	0.32 (0.04–1.16)	0.97 (0.36–2.09)	1.62 (0.53–3.75)	0.32 (0.01–1.77)
N/A	0.64 (0.18–1.64)	6.60 (4.78–8.85)	6.49 (4.01–9.85)	6.71 (4.20–10.07)
Siblings				
No siblings	81.32 (78.03–84.31)	85.99 (83.01–88.62)	85.06 (80.59–88.85)	86.90 (82.65–90.43)
1+ siblings	17.87 (14.94–21.12)	14.01 (11.38–16.99)	14.94 (11.15–19.41)	13.10 (9.57–17.35)
N/A	0.81 (0.26–1.87)	-	-	-
Family Wealth Index^a^				
Low	27.38 (23.90–31.06)	-	-	-
Medium	28.99 (25.44–32.73)	-	-	-
High	23.51 (20.23–27.05)	-	-	-
N/A	20.13 (17.04–23.50)	-	-	-

N/A indicates missing values.

“-” indicates not applicable.

a Family wealth index was developed based on responses for GEAS baseline surveys administered from November to December 2017. All the rest demographic factors from the pre-COVID period were measured at the GEAS Wave 2 interview conducted from November to December 2018.

### Concerns about COVID-19 exposure

Boys and girls reported distinct perceptions and COVID-19-related experiences (Table 2). Girls were more likely to report feeling threatened or very concerned about COVID-19 than boys (50.8% vs. 41.9%, respectively, *p* < .01). Concerns about transmission emerged prominently in the girls’ focus groups discussions:

*“I worried that everything I touched was not very safe. I have the plastic teeth braces, I take them off every time before I eat food, and then I wear them back after eating. I often worry that the braces and my hands are not clean. In fact, the braces are plastic and cannot be scalded with boiling water. However, I was scared, so I scalded them with boiling water, and then I could not wear them. Then my mother scolded me.” (Girl aged 14–15).*

**Table 2:** Sex-differences in COVID-19 concerns, economic and educational implications, and health indicators during COVID-19 among adolescents from the GEAS study in Shanghai, China

Concerns and social, economic, and educational implications of COVID	All (N = 621) % (95% CI)	Boys (N = 308) % (95% CI)	Girls (N = 313) % (95% CI)	*p* value^b^
Concerned about COVID pandemic				
Yes (Mean score ≥ sample mean)	46.38 (42.40–50.39)	41.88 (36.31–47.61)	50.80 (45.12–56.47)	*.008*
N/A	2.09 (1.12–3.55)	1.95 (0.72–4.19)	2.24 (0.90–4.55)	
Jobs loss/income reduction in family				
Yes (Agree a little/a lot)	28.66 (25.14–32.40)	32.14 (26.96–37.67)	25.24 (20.52–30.43)	*.041*
N/A	1.45 (0.66–2.73)	2.27 (0.92–4.63)	0.64 (0.08–2.29)	
Family had difficulty affording things				
Yes (Agree a little/a lot)	10.79 (8.46–13.50)	12.99 (9.44–17.26)	8.63 (5.76–12.30)	*.073*
N/A	1.13 (0.45–2.31)	1.62 (0.53–3.75)	0.64 (0.08–2.29)	
Food insecurity				
Yes (Sometimes/often)	7.57 (5.61–9.94)	10.39 (7.22–14.35)	4.79 (2.71–7.78)	*.009*
N/A	2.25 (1.24–3.75)	1.95 (0.72–4.19)	2.56 (1.11–4.97)	
Concerned about current grade completion				
Yes (Somewhat/very concerned)	52.82 (48.81–56.80)	45.13 (39.48–50.87)	60.38 (54.73–65.84)	*<.001*
N/A	1.77 (0.89–3.15)	2.60 (1.13–5.05)	0.96 (0.20–2.78)	

Health behaviors	Mean (95% CI)	Mean (95% CI)	Mean (95% CI)	*p* value
Length of sleep during COVID remote learning (hours)^a^	8.93 (8.84–9.03)	9.05 (8.90–9.20)	8.81 (8.70–8.92)	*.011*
Averaged number of physical activities in the past week (hours/day)	1.16 (1.07–1.25)	1.39 (1.24–1.55)	0.90 (0.82–0.99)	*<.001*

Health outcomes	% (95% CI)	% (95% CI)	% (95% CI)	*p* value
Overall health				
Poor/Fair	13.37 (10.79–16.30)	9.09 (6.13–12.87)	17.57 (13.52–22.25)	
Good/Excellent	85.83 (82.84–88.48)	89.94 (86.02–93.06)	81.79 (77.06–85.91)	*.002*
N/A	0.81 (0.26–1.87)	0.97 (0.20–2.82)	0.64 (0.08–2.29)	
Depressive symptoms				
Mean (95% CI)	2.62 (2.53–2.71)	2.52 (2.39–2.65)	2.72 (2.59–2.84)	*.030*
Generalized Anxiety Disorder (GAD-7)				
No	61.19 (57.23–65.04)	65.85 (59.99–70.88)	56.87 (51.18–62.43)	
Mild	24.32 (20.99–27.89)	23.38 (18.76–28.51)	25.24 (20.52–30.43)	
Moderate	5.96 (4.23–8.12)	4.55 (2.51–7.51)	7.35 (4.71–10.82)	*.034*
Severe	6.12 (4.37–8.30)	4.55 (2.51–7.51)	7.67 (4.97–11.19)	
N/A	2.42 (1.36–3.95)	1.95 (0.72–4.19)	2.88 (1.32–5.39)	

Two-sided *P* < .05 is considered statistical significance and indicated in italic. N/A indicates missing values.

a Outliers (n = 45) of averaged sleep hours for remote learning phases were excluded from the calculation.

b *p* values were calculated among adolescents with complete information on the indicators.

### Gendered concerns related to the impacts of the COVID-19 pandemic

While boys and girls alike reflected on the social consequences of COVID-19 lockdowns, boys reported greater concern regarding the economic consequences of familial job loss, with one-third of boys versus one-quarter of girls indicating family loss of income (*p* = .04). Boys were also more likely to indicate instances of food shortages (10.4% vs. 4.8%, *p* < .01); this was also reflected in the focus group discussions:

*“I was afraid that foods in the supermarket and the vegetable market would be sold out, and my family could'nt buy food and daily essentials.” (Boy aged 16–18)*

On the other hand, girls expressed greater concerns than boys regarding their school performance because of the lockdown (60.4% vs. 45.1%, respectively, *p* < .001). These concerns were echoed in the focus group discussions among older girls who shared their considerations about switching from the STEM-focused curriculum (science, technology, engineering, and mathematics) to the art-focused curriculum, which was perceived as less challenging due to fears of academic underperformance.

*“No, it's a compromised plan to take the art test. It needs to have a specialty, but if somebody has a high score on general subjects, who wants to compete on a specialty?” (Girl aged 16–18)*

### Gendered health outcomes during the COVID-19 pandemic

Sex differences in health-related outcomes were also evident. Girls were less likely than boys to rate their general health as good or excellent (81.8% vs. 89.9%, *p* < .01) and, on average, reported greater depressive symptoms (mean scores of 2.7 vs. 2.5 on a scale of 1–5, *p* = .03). Girls were also more likely than boys to report moderate to severe anxiety symptoms (43.1% vs. 34.1%, *p* = .03).

In focus group discussions, girls related their psychological distress to their education and lack of peer interactions, while boys described school closures more positively.

*“The good thing was that we did'nt need to go out, however, I would not consciously work hard on the study and the efficiency would decline. (Girl aged 16–18)”*

*“At that time, the social circle was basically limited to family members and friends on the Internet. I always felt that I might be a little bit out of touch with reality. So, I thought there may be some psychological changes.” (Girl aged 16–18)*

*“The happiest thing was that we could take an online class. The schedule was relatively flexible. We could arrange the learning tasks according to our needs and habits, what’s more, there was less homework in online class.” (Boy aged 14–15)*

*“Interviewer: Will there be any psychological problems?*

*Respondent: ‘There is no psychological problem, because every Friday at 1:15**p.m.**, our psychological teacher will give us psychological counseling.’” (Boy aged 14–15)*

### Changes in health-related outcomes before and during the COVID-19 pandemic

**Figure 1:**
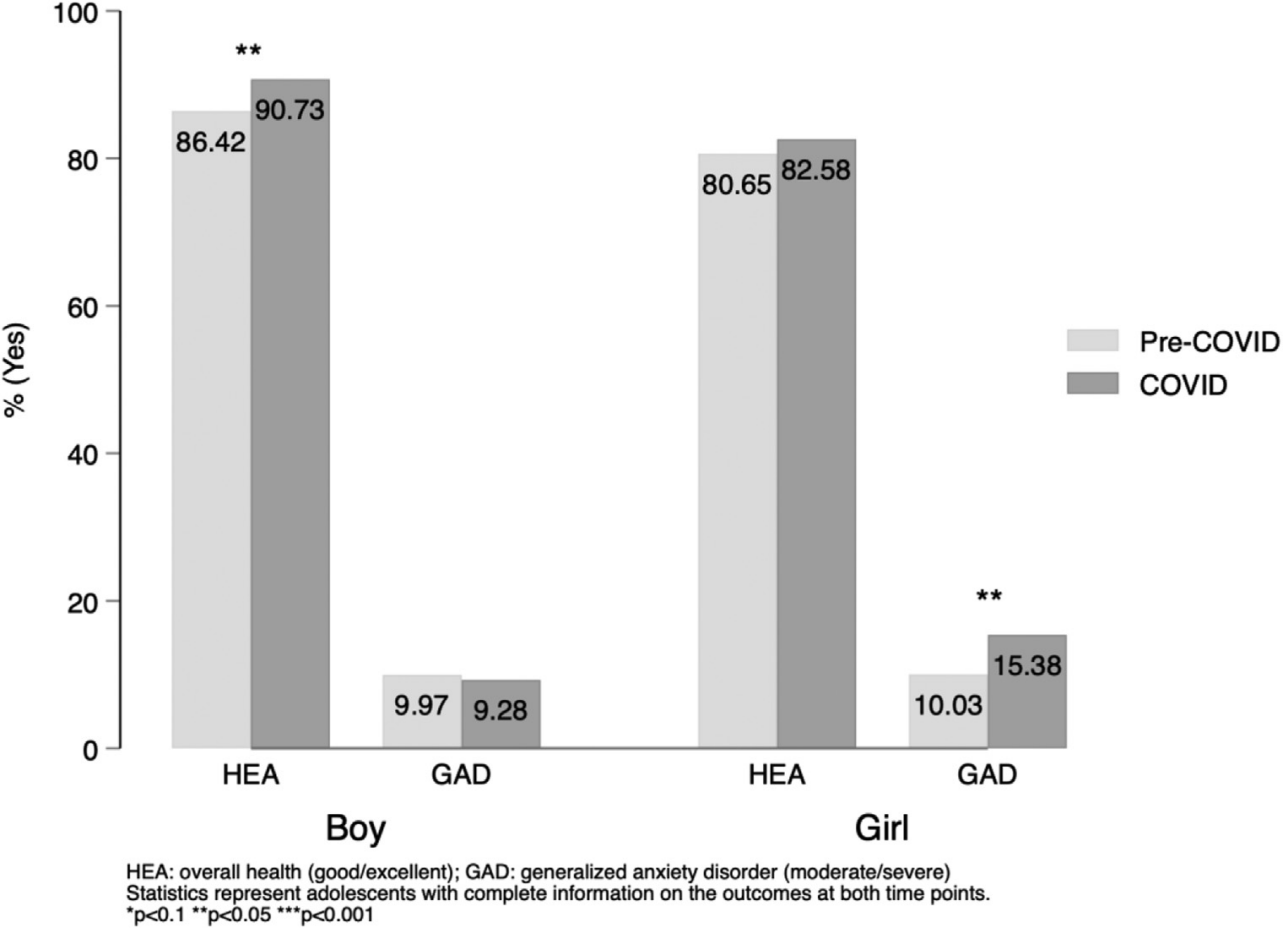
Trends in overall health and generalized anxiety disorder among adolescents from Shanghai, China before and postpandemic.

As sex differences may have predated COVID-19, we investigated how general health perceptions and mental health indicators changed between pre-COVID-19 and COVID-19 time points (Supplementary Table 1). Comparing measures between pre-COVID-19 and COVID-19 surveys for the same adolescents, we found that boys’ perceptions of their health improved during the pandemic (from 86.4% to 90.7% reporting good/excellent health), but that girls’ perceptions remained unchanged (from 80.7% to 82.6%) (Figure 1). These findings were confirmed in the regression analysis (Table 3).

**Table 3:** Unadjusted time effects on overall health, depressive symptoms, and generalized anxiety disorder and COVID-related experience effect modification on these time effects

Overall health (good/excellent)	All		Boys		Girls	
	OR (95% CI)	*p* value	Or (95% CI)	*p* value	OR (95% CI)	*p* value
Unadjusted time effect (trend)	1.28 (1.00, 1.64)	.05	**1.54 (1.00, 2.36)**	**.05**	1.14 (0.83, 1.55)	.41
Time effect × reduction in family income						
[interaction - coefficient (95% CI)]	−0.37 (−0.90, 0.16)	.18	***−0.95 (−1.86, −0.04)***	***.04***	0.01 (−0.67, 0.68)	.99
Time effect among adolescents with no income loss	**1.44 (1.06, 1.96)**	**.02**	**2.10 (1.24, 3.58)**	**.01**	1.14 (0.78, 1.67)	.51
Time effect among adolescents with Income loss	1.00 (0.65, 1.54)	1.00	0.81 (0.39, 1.70)	.59	1.14 (0.66, 1.99)	.63

Depressive symptoms	All		Boys		Girls	
	Change in mean score (95% CI)	*p* value	Change in mean score (95% CI)	*p* value	Change in mean score (95% CI)	*p* value
Unadjusted time (effect)	**−0.21 (−0.30, −0.11)**	**<.001**	**−0.25 (−0.40, −0.10)**	**.001**	**−0.17 (−0.29, −0.04)**	**.01**
Time effect × concerned about COVID pandemic						
[interaction - coefficient (95% CI)]	0.11 (−0.10, 0.30)	.29	0.19 (−0.12, 0.49)	.23	0.01 (−0.24, 0.27)	.91
Time effect among adolescents who are not concerned about COVID (mean score < sample mean)	**0.26 (−0.39, −0.12)**	**<.001**	**−0.33 (−0.52, −0.13)**	**.001**	−0.17 (−0.35, 0.01)	.06
Time effect among adolescents who are concerned about COVID (mean score ≥ sample mean)	**−0.15 (−0.29, −0.01)**	**.04**	−0.14 (−0.37, 0.09)	.24	−0.16 (−0.34, 0.02)	.08
Time effect × concerned about grade completion						
[interaction - coefficient (95% CI)]	***0.27 (0.07, 0.46)***	***.01***	***0.31 (0.01, 0.61)***	***.04***	0.21 (−0.05, 0.47)	.11
Time effect among adolescents who are not concerned about grade	**−0.35 (−0.50, −0.21)**	**<.001**	**−0.40 (−0.60, −0.19)**	**<.001**	**−0.29 (−0.49, −0.09)**	**<.01**
Time effect among adolescents who are concerned about grade	−0.09 (−0.22, 0.05)	.21	−0.09 (−0.30, 0.13)	.43	−0.08 (−0.24, 0.08)	.30
Time effect × food insecurity						
[interaction - coefficient (95% CI)]	***0.45 (0.08, 0.81)***	***.02***	***0.72 (0.23, 1.20)***	***.004***	−0.037 (−0.63, 0.55)	.90
Time effect among adolescents who have no food insecurity	**−0.24 (−0.34, −0.14)**	**<.001**	**−0.32 (−0.48, −0.17)**	**<.001**	**−0.16 (−0.29, −0.03)**	**.01**
Time effect among adolescents who have food insecurity	0.21 (−0.15, 0.56)	.25	0.40 (−0.06, 0.85)	.09	−0.20 (−0.77, 0.37)	.50

Generalized anxiety disorder (moderate or severe)	All		Boys		Girls	
	OR (95% CI)	*p* value	OR (95% CI)	*p* value	OR (95% CI)	*p* value
Unadjusted Time effect (trends)	1.27 (0.92, 1.75)	.14	0.92 (0.55, 1.54)	.76	**1.63 (1.09, 2.45)**	**.02**
Time effect × concerned about grade completion						
[interaction - coefficient (95% CI)]	***0.76 (0.08, 1.43)***	***.03***	0.80 (−0.24, 1.84)	.13	0.49 (−0.44, 1.44)	.30
Time effect among adolescents who are not concerned about grade	0.78 (0.46, 1.34)	.38	0.62 (0.30, 1.30)	.21	1.14 (0.51, 2.55)	0.76
Time effect among adolescents who are concerned about grade	1.67 (1.11, 2.50)	0.01	1.38 (0.66, 2.87)	.39	**1.85 (1.16, 2.97)**	**.01**

Unadjusted trends referred to the changes in each outcome measured during COVID compared to the pre-COVID period among adolescents and among boys and girls, respectively.

Effect modification is demonstrated by significant interactions highlighted in bold and italic font.

However, we also found that trends in health indicators varied according to the pandemic’s economic impact (Table 3). Specifically, boys who were economically affected by COVID-19 experienced no change in their health status (OR: 0.81, 95% CI: 0.39, 1.70), while those who were not economically affected perceived a significant rise in good health (OR: 2.10, 95% CI: 1.24, 3.58). Similar trends were not observed for girls.

**Figure 2:**
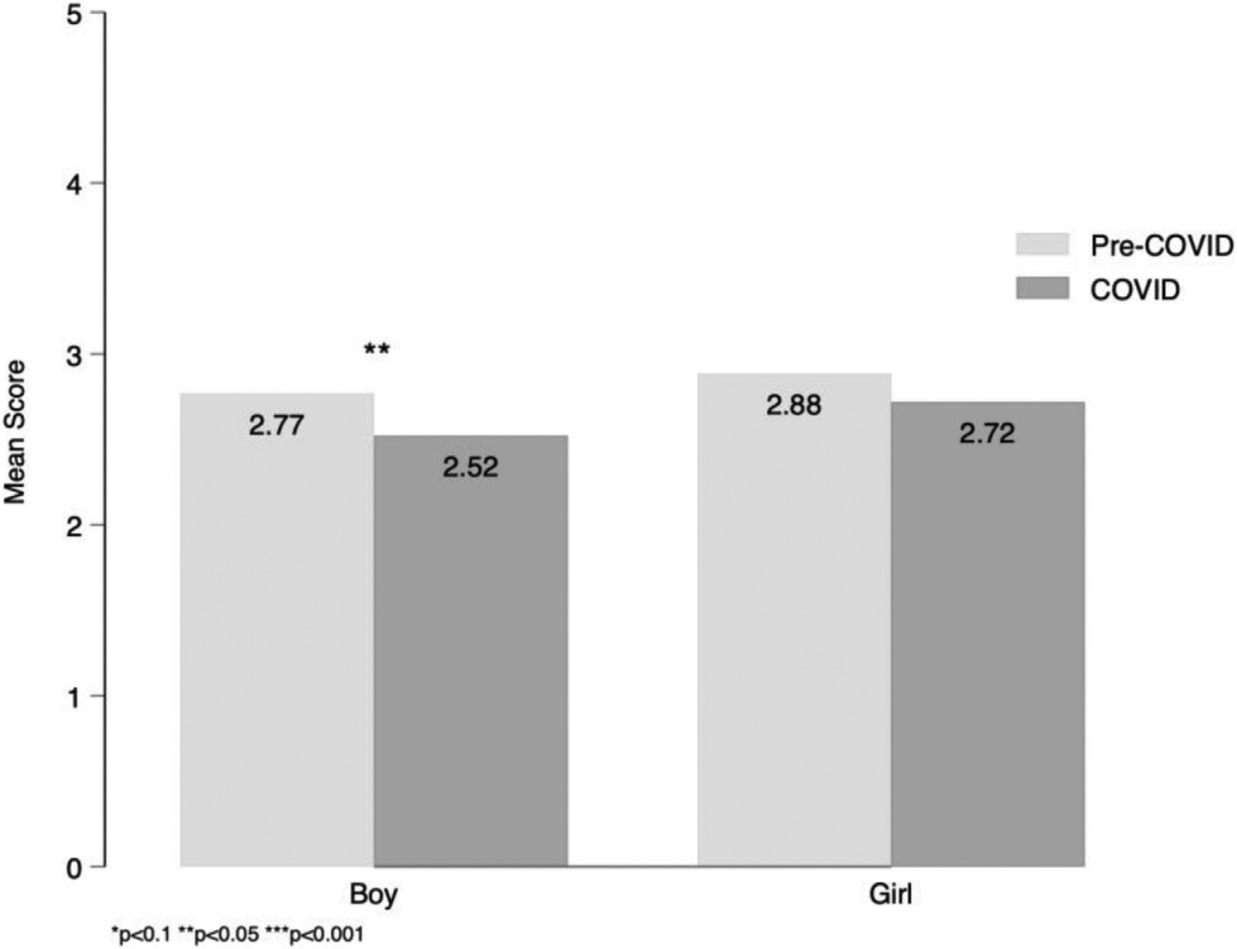
Trends of depressive symptoms among adolescents from Shanghai, China before and postpandemic.

Sex differences were also noted for depressive symptoms and anxiety (Figures 1 and 2), which remained stable for boys but increased in the case of anxiety for girls (OR: 1.63, 95% CI: 1.09, 2.45) (Table 3). Boys and girls experienced declines in depressive symptomatology during COVID-19; however, there were sex differences as to what accounted for the decline in depressive symptoms. Specifically, for boys but not for girls (Table 3), those who were confident about completing their school grades or did not experience food shortages reported fewer depressive symptoms during COVID-19 compared to the pre-COVID-19 time point (decrease in the mean score: −0.40, 95% CI: −0.60, −0.19; −0.32, 95% CI: −0.48, −0.17 respectively). Girls who were concerned about grade completion reported increased levels of anxiety (OR: 1.85, 95% CI: 1.16, 2.97), while this was not the case for girls without such concerns (OR: 1.14, 95% CI: 0.51, 2.55). Adolescent boys reported nonsignificant declines in anxiety when they had no concerns about school progression (OR: 0.62, 95% CI: 0.30, 1.30) and no significant increases when they reported school concerns (OR: 1.38, 95% CI: 0.66, 2.87). Complete results of trends in health-related outcomes stratified by COVID-19 experiences are presented in Supplementary Tables 2–4.

## Discussion

This study conducted among low-income, urban adolescents in Shanghai offers insights into adolescents’ gendered experiences related to the COVID-19 pandemic. Our findings illustrate the ways the pandemic has magnified gender and social disparities in this population while also drawing a nuanced picture of the impact of COVID-19 on adolescent health. Consistent with prior research in China [22] and other studies in high-income countries [23], adolescents in our study perceived health threats related to COVID-19 and concerns for their education and family’s economic stability. Girls emphasized the educational and health implications of COVID-19, while boys focused more on financial stability.

Gender differences in adolescents’ perceptions of COVID-19 health threats, where girls expressed more significant concerns than boys, are consistent with prior studies in Europe [23,24]. These perceived threats may encourage young people to comply with COVID-19 preventive measures, but they appear to also increase adolescents’ psychological distress [25,26]. Specifically, we found that boys who perceived limited health threats from COVID-19 experienced fewer depressive symptoms between the pre-COVID-19 and COVID-19 periods, while those who perceived greater threats experienced no improvement, confirming results from two cross-sectional studies in China reporting increased mental health problems among adolescents who perceived greater COVID-19 health threats [26,27].

Despite resuming in-person classes after 2 months of lockdown, adolescents in our study expressed heightened concerns about their educational achievement. Consistent with prior research [23,24], girls were more likely to worry about their education than boys, leading them to consider less rigorous educational tracks to avoid failing their general exams. These results complement emerging research that sounds the alarm about the consequences of school closure for adolescents’ educational trajectories [8,28] and psychosocial health [8,29]. Beyond school dropouts, our study points to the importance of investigating levels and determinants of curriculum shifts, as these are likely to have long-term consequences on professional development and contribute to social and gender disparities.

Gendered patterns were also seen in adolescents’ assessment of the financial consequences of COVID-19, as boys in our study expressed more concerns about their family’s economic stability and reported greater food insecurity than girls. A study in Spain among participants aged 6–25 found opposite sex differences [23]; together, these findings raise attention to the COVID-19-induced economic disruptions in young people’s lives. Our study was fielded in June 2020, by which time the Chinese economy had already begun to recover, while the study conducted by colleagues Muñoz-Fernández et al. was carried out during the lockdown period. Differences in findings may also reflect the benefits of China’s social welfare expansion, which has provided additional direct assistance to low-income families since the start of the pandemic [30]. While these measures may have eased the effect of the economic downturn, our results still draw attention to the economic implications of the pandemic on health inequities, as trends in mental health and overall self-rated health differed between boys who reported economic hardships and those who did not. These findings align with the results of two cross-sectional studies, showing increased depressive symptoms among adolescents reporting family financial strain during COVID-19 in the United States [31] or food insecurity in China [32].

The observed sex differences in the manifestation of COVID-19 related concerns draw attention to ingrained stereotypical gender roles, with males expressing concerns about failing to fulfill their duties as “providers,” as well as to growing health disparities [33]. The worsening of girls’ anxiety during COVID-19 is consistent with prior research, but boys’ improved self-perceived health and mental health is a surprising finding. Comparisons across studies are challenging, given differences in the timing of surveys, sample selection, methods of assessment, and study design. Cross-sectional studies comparing COVID-19 estimates to expected pre-COVID-19 levels or directly asking respondents to assess the impact of COVID-19 on their health generally report a rise in depression and anxiety during COVID-19 [22,26,[34], [35], [36]]. Studies also find more significant psychosocial distress in quarantine relative to nonquarantine conditions [37] and heightened levels among girls than boys [22,36,38]. However, few longitudinal studies have examined individual trends in health indicators between pre-COVID-19 and COVID-19 conditions, limiting actual rather than a perceived assessment of COVID-19’s impact. Three longitudinal studies suggest rising depressive symptoms during COVID-19 with inconsistent trends regarding anxiety [34,38,39], but none report on the differential gender trends we observed in our study. Based on boys’ narratives, we hypothesize that the observed improvement in boys’ wellbeing may be due to reduced academic pressure, a dimension of adolescents’ lives found to increase psychological distress in China [40]. However, diminished school pressure does not seem to benefit girls in that some appear to change their educational strategies (such as taking less rigorous curricula), reflecting differential adaptation to the pandemic. Based on the data presented here, programs to address adolescent experiences during COVID-19 must recognize and intervene in the distinct ways that boys and girls feel and manage concerns related to the virus.

While this study provides unique longitudinal insights on the gendered experiences of low-income urban young adolescents during the COVID-19 pandemic in Shanghai, our investigation is not without limitations. The GEAS convenience sample limits the generalizability of the findings to other populations, including rural populations in China, which have experienced a significant impact of economic hardship induced by COVID-19 [41]. In addition, approximately 9%–13% of adolescents who completed the COVID-19 interview were excluded from the analyses due to poor data quality (i.e., high missingness in survey responses) or lack of data on key outcomes at either time point. However, we conducted a sensitivity analysis to understand the potential for bias in our sample selection, comparing characteristics of adolescents included versus excluded in the analysis. Results indicate similarities in the characteristics between groups, both demographically and in terms of most health symptoms explored in this analysis, limiting concerns about bias. Additionally, the study does not use a diagnostic measure of depression, although the use of the same indicators in the pre-COVID-19 and COVID-19 surveys allow for trends analysis, regardless of the overall prevalence of depressive symptomology explored. In the absence of a “control” group of adolescents who were not exposed to COVID-19, it is difficult to assess how much of the observed trends are related to COVID-19 or reflect secular trends of aging adolescents. However, a subanalysis comparing adolescents who were interviewed in January 2020 to those who were interviewed in June 2020 shows similar gender differences in adolescents’ health trends during COVID-19, suggesting much of the reported trends are related to the pandemic.

### Conclusion

This study, carried out in the immediate aftermath of the 2020 COVID-19 lockdown in China, suggests adolescents’ COVID-19 experiences are highly gendered in Shanghai, as girls are more concerned about the pandemic’s implications for their health and education, while boys perceive greater economic threats. Gender disparities also emerge with respect to adolescents’ general and mental health, as boys reported improvements while girls suffered from increased anxiety leading to poorer general health.
