# Supplementary material for: COVID-19 Experiences and Health-Related Implications: Results From a Mixed-Method Longitudinal Study of Urban Poor Adolescents in Shanghai
Source: J Adolesc Health. 2022 Jul;71(1):30–8. doi: 10.1016/j.jadohealth.2022.03.016 (PMC9077362; doi:10.1016/j.jadohealth.2022.03.016)
Supplement: Supplementary Table 1 [file mmc1.docx]

Supplementary Table 1: Distributions of health-related outcomes during the COVID pandemic compared to the pre-COVID period among all adolescents and boys and girls separately

|  | All | | Boys | | Girls | |
| --- | --- | --- | --- | --- | --- | --- |
|  | Pre-COVID | COVID | Pre-COVID | COVID | Pre-COVID | COVID |
| **Overall Health, % (95% CI)** |  |  |  |  |  |  |
| Poor/Fair | 16.5  (13.65 - 19.69) | 13.40  (10.80 - 16.36) | 13.58  (9.92 - 17.96) | 9.27  (6.25 - 13.12) | 19.35  (15.11 - 24.20) | 17.42  (13.37 - 22.11) |
| Good/Excellent | 83.50  (80.31 - 86.35) | 86.60  (83.64 - 89.20) | 86.42  (82.04 - 90.08) | 90.73  (86.88 - 93.75) | 80.65  (75.80 - 84.89) | 82.58  (77.89 - 86.63) |
| **Depressive Symptoms** |  |  |  |  |  |  |
| Mean (95% CI) | 2.83  (2.74 - 2.92) | 2.62  (2.53 - 2.71) | 2.77  (2.63 - 2.90) | 2.52  (2.39 - 2.65) | 2.88  (2.76 - 3.01) | 2.72  (2.60 - 2.84) |
| **GAD, % (95% CI)** |  |  |  |  |  |  |
| No/Mild | 90.00  (87.29 - 92.30) | 87.63  (84.70 - 90.17) | 90.03  (86.00 - 93.22) | 90.72  (86.79 - 93.80) | 89.97  (85.99 - 93.13) | 84.62  (80.02 - 88.51) |
| Moderate/Severe | 10.00  (7.70 - 12.71) | 12.37  (9.83 - 15.30) | 9.97  (6.78 - 14.00) | 9.28  (6.20 - 13.21) | 10.03  (6.87 - 14.01) | 15.38  (11.49 - 19.98) |

*GAD: generalized anxiety disorder.*

*Note: Calculations are based on observations with complete outcome information at both time points.*
