# Supplementary material for: COVID-19 Experiences and Health-Related Implications: Results From a Mixed-Method Longitudinal Study of Urban Poor Adolescents in Shanghai
Source: J Adolesc Health. 2022 Jul;71(1):30–8. doi: 10.1016/j.jadohealth.2022.03.016 (PMC9077362; doi:10.1016/j.jadohealth.2022.03.016)
Supplement: Supplementary Table 2 [file mmc2.docx]

Supplementary Table 2: Changes in perceived overall health during COVID pandemic compared to the pre-COVID period among all participants and by sex under different COVID impacts

| **Overall Health** | **All** | | **Boys** | | **Girls** | |
| --- | --- | --- | --- | --- | --- | --- |
|  | **OR (95% CI)** | **P-value** | **OR (95% CI)** | **P-value** | **OR (95% CI)** | **P-value** |
| **Job loss within the family** |  |  |  |  |  |  |
| [interaction - coefficient (95% CI)] | -0.367 (-0.898, 0.164) | 0.175 | ***-0.950 (-1.861, -0.040)*** | ***0.041*** | 0.005 (-0.666, 0.676) | 0.988 |
| No | **1.44 (1.06, 1.96)** | **0.019** | **2.10 (1.24, 3.59)** | **0.006** | 1.14 (0.78, 1.67) | 0.509 |
| Yes | 1.00 (0.65, 1.54) | 1.000 | 0.81 (0.39, 1.70) | 0.584 | 1.14 (0.66, 1.99) | 0.633 |
| **Concerned about COVID pandemic** |  |  |  |  |  |  |
| [interaction - coefficient (95% CI)] | -0.196 (-0.699, 0.306) | 0.443 | -0.196 (-1.060, 0.667) | 0.656 | -0.188 (-0.811, 0.434) | 0.553 |
| No | 1.41 (0.99, 2.03) | 0.060 | 1.70 (0.92, 3.14) | 0.091 | 1.26 (0.80, 1.98) | 0.323 |
| Yes | 1.16 (0.82, 1.65) | 0.397 | 1.40 (0.76, 2.56) | 0.281 | 1.04 (0.68, 1.59) | 0.852 |
| **Concerned about grade completion** |  |  |  |  |  |  |
| [interaction - coefficient (95% CI)] | -0.038 (-0.539, 0.464) | 0.883 | -0.119 (-0.976, 0.738) | 0.786 | 0.111 (-0.532, 0.753) | 0.736 |
| No | 1.31 (0.90, 1.90) | 0.166 | 1.62 (0.91, 2.90) | 0.101 | 1.06 (0.64, 1.77) | 0.821 |
| Yes | 1.26 (0.90, 1.75) | 0.175 | 1.44 (0.77, 2.71) | 0.257 | 1.18 (0.80, 1.75) | 0.393 |
| **Food insecurity** |  |  |  |  |  |  |
| [interaction - coefficient (95% CI)] | -0.576 (-1.429, 0.277) | 0.186 | -1.150 (-2.498, 0.198) | 0.095 | -0.140 (-1.351, 1.071) | 0.821 |
| No | **1.35 (1.04, 1.75)** | **0.025** | **1.76 (1.12, 2.78)** | **0.015** | 1.15 (0.83, 1.59) | 0.399 |
| Yes | 0.76 (0.34, 1.71) | 0.505 | 0.56 (0.16, 1.98) | 0.366 | 1.00 (0.31, 3.21) | 1.000 |
