# Supplementary material for: COVID-19 Experiences and Health-Related Implications: Results From a Mixed-Method Longitudinal Study of Urban Poor Adolescents in Shanghai
Source: J Adolesc Health. 2022 Jul;71(1):30–8. doi: 10.1016/j.jadohealth.2022.03.016 (PMC9077362; doi:10.1016/j.jadohealth.2022.03.016)
Supplement: Supplementary Table 3 [file mmc3.docx]

Supplementary Table 3: Changes in depressive symptoms during the COVID pandemic compared to the pre-COVID period among all participants and by sex under different COVID impacts

| **Depressive Symptoms** | **All** | | **Boys** | | **Girls** | |
| --- | --- | --- | --- | --- | --- | --- |
|  | **Mean Score (95% CI)** | **P-value** | **Mean Score (95% CI)** | **P-value** | **Mean Score (95% CI)** | **P-value** |
| **Job loss within family** |  |  |  |  |  |  |
| [interaction - coefficient (95% CI)] | 0.200 (-0.015, 0.416) | 0.069 | 0.296 (-0.023, 0.614) | 0.069 | 0.108 (-0.181, 0.398) | 0.464 |
| No | **-0.26 (-0.38, -0.15)** | **<0.001** | **-0.34 (-0.52, -0.16)** | **<0.001** | **-0.19 (-0.34, -0.05)** | **0.010** |
| Yes | -0.06 (-0.25, 0.12) | 0.493 | -0.05 (-0.31, 0.22) | 0.725 | -0.08 (-0.33, 0.17) | 0.508 |
| **Concerned about COVID pandemic** |  |  |  |  |  |  |
| [interaction - coefficient (95% CI)] | 0.105 (-0.091, 0.301) | 0.293 | 0.186 (-0.116, 0.489) | 0.227 | 0.014 (-0.238, 0.266) | 0.914 |
| No | **-0.26 (-0.39, -0.12)** | **<0.001** | **-0.33 (-0.52, -0.13)** | **0.001** | -0.17 (-0.35, 0.01) | 0.061 |
| Yes | **-0.15 (-0.29, -0.01)** | **0.041** | -0.14 (-0.37, 0.09) | 0.235 | -0.16 (-0.34, 0.02) | 0.080 |
| **Concerned about grade completion** |  |  |  |  |  |  |
| [interaction - coefficient (95% CI)] | ***0.266 (0.071, 0.461)*** | ***0.008*** | ***0.309 (0.012, 0.607)*** | ***0.041*** | 0.208 (-0.050, 0.465) | 0.114 |
| No | **-0.35 (-0.50, -0.21)** | **<0.001** | **-0.40 (-0.60, -0.19)** | **<0.001** | **-0.29 (-0.49, -0.09)** | **0.004** |
| Yes | -0.09 (-0.22, 0.05) | 0.206 | -0.09 (-0.30, 0.13) | 0.432 | -0.08 (-0.24, 0.08) | 0.304 |
| **Food insecurity** |  |  |  |  |  |  |
| [interaction - coefficient (95% CI)] | ***0.446 (0.078, 0.813)*** | ***0.017*** | ***0.718 (0.23, 1.20)*** | ***0.004*** | -0.037 (-0.625, 0.552) | 0.903 |
| No | **-0.24 (-0.34, -0.14)** | **<0.001** | **-0.32 (-0.48, -0.17)** | **<0.001** | **-0.16 (-0.29, -0.03)** | **0.013** |
| Yes | 0.21 (-0.15, 0.56) | 0.254 | 0.40 (-0.06, 0.85) | 0.090 | -0.20 (-0.77, 0.37) | 0.495 |
