# Supplementary material for: COVID-19 Experiences and Health-Related Implications: Results From a Mixed-Method Longitudinal Study of Urban Poor Adolescents in Shanghai
Source: J Adolesc Health. 2022 Jul;71(1):30–8. doi: 10.1016/j.jadohealth.2022.03.016 (PMC9077362; doi:10.1016/j.jadohealth.2022.03.016)
Supplement: Supplementary Table 4 [file mmc4.docx]

Supplementary Table 4: Changes in generalized anxiety disorder during the COVID pandemic compared to the pre-COVID period among all participants and by sex under different COVID impacts

| **Generalized Anxiety Disorder (moderate or severe)** | **All** | | **Boys** | | **Girls** | |
| --- | --- | --- | --- | --- | --- | --- |
|  | **OR (95% CI)** | **P-value** | **OR (95% CI)** | **P-value** | **OR (95% CI)** | **P-value** |
| **Job loss within family** |  |  |  |  |  |  |
| [interaction - coefficient (95% CI)] | 0.025 (-0.652, 0.703) | 0.942 | -0.058 (-1.142, 1.027) | 0.917 | 0.108 (-0.773, 0.988) | 0.811 |
| No | 1.26 (0.85, 1.86) | 0.246 | 0.94 (0.50, 1.77) | 0.853 | 1.58 (0.95, 2.61) | 0.075 |
| Yes | 1.29 (0.74, 2.25) | 0.362 | 0.89 (0.37, 2.15) | 0.795 | 1.76 (0.85, 3.62) | 0.127 |
| **Concerned about COVID pandemic** |  |  |  |  |  |  |
| [interaction - coefficient (95% CI)] | -0.104 (-0.749, 0.541) | 0.752 | -0.648 (-1.682, 0.387) | 0.220 | 0.221 (-0.619, 1.061) | 0.605 |
| No | 1.34 (0.84, 2.16) | 0.220 | 1.26 (0.62, 2.55) | 0.530 | 1.44 (0.76, 2.71) | 0.262 |
| Yes | 1.21 (0.78, 1.88) | 0.390 | 0.66 (0.31, 1.39) | 0.274 | **1.79 (1.04, 3.11)** | **0.037** |
| **Concerned about grade completion** |  |  |  |  |  |  |
| [interaction - coefficient (95% CI)] | ***0.756 (0.081, 1.431)*** | ***0.028*** | 0.799 (-0.243, 1.841) | 0.133 | 0.491 (-0.444, 1.425) | 0.304 |
| No | 0.78 (0.46, 1.34) | 0.376 | 0.62 (0.30, 1.30) | 0.205 | 1.14 (0.51, 2.55) | 0.758 |
| Yes | 1.67 (1.11, 2.51) | 0.013 | 1.38 (0.66, 2.87) | 0.393 | **1.85 (1.16, 2.97)** | **0.010** |
| **Food insecurity** |  |  |  |  |  |  |
| [interaction - coefficient (95% CI)] | 0.347 (-0.763, 1.456) | 0.540 | 0.952 (-0.799, 2.702) | 0.287 | -0.118 (-1.598, 1.362) | 0.876 |
| No | 1.23 (0.88, 1.72) | 0.221 | 0.84 (0.49, 1.44) | 0.522 | **1.65 (1.08, 2.52)** | **0.020** |
| Yes | 1.74 (0.60, 5.02) | 0.304 | 2.17 (0.41, 11.46) | 0.362 | 1.47 (0.36, 6.06) | 0.596 |
